# Tetraether biomarker records from a loess-paleosol sequence in the western Chinese Loess Plateau

**DOI:** 10.3389/fmicb.2013.00199

**Published:** 2013-07-15

**Authors:** Guodong Jia, Zhiguo Rao, Jie Zhang, Zhiyang Li, Fahu Chen

**Affiliations:** ^1^State Key Laboratory of Organic Geochemistry, Guangzhou Institute of Geochemistry, Chinese Academy of SciencesGuangzhou, China; ^2^Key Laboratory of Western China's Environmental Systems (Ministry of Education), Lanzhou UniversityLanzhou, China

**Keywords:** GDGTs, archaea, bacteria, Chinese Loess Plateau, paleoclimate proxies

## Abstract

The ubiquitous occurrence of glycerol dialkyl glycerol tetraethers (GDGTs) in soils and their ability to record temperature and environmental changes offer the prospect of independently reconstructing continental paleotemperature and paleoenvironment from the loess-paleosol sequences (LPS) from the Chinese Loess Plateau (CLP). In this study we present records of GDGT-derived proxies for the last 70 kyr from the Yuanbao LPS, western CLP. Temperature record reconstructed from the cyclization and methylation index of branched tetraethers (MBT-CBT) displays that the onset of deglacial warming at ~20 kyr before present (BP) precedes the strengthening of summer monsoon at ~15 kyr BP, which is in agreement in timing with previous MBT-CBT temperature records from the southeastern CLP. The maximal deglacial warming of ~10°C is slightly higher than those in the southeastern CLP, perhaps due to the higher latitude and farther inland of the study site. The Branched and Isoprenoid Tetraether (BIT) index shows higher values (0.87–0.96 range, 0.93 average) in the glacial loess and lower values (0.76–0.91 range, 0.83 average) in the Holocene paleosols, with a steady decreasing trend since the early Holocene. The decreasing trend could suggest enhanced Thaumarchaeota relative to GDGT producing bacteria activity since the early Holocene, but other possibilities, such as preferential degradation of isoprenoid GDGTs or upward increase in living archaea relative to bacteria in the paleosol profile, cannot be fully excluded. Our results thus demonstrate the need of future study on microbial community structure in soil column and differential degradation of GDGT molecules.

## Introduction

**Figure 1:**
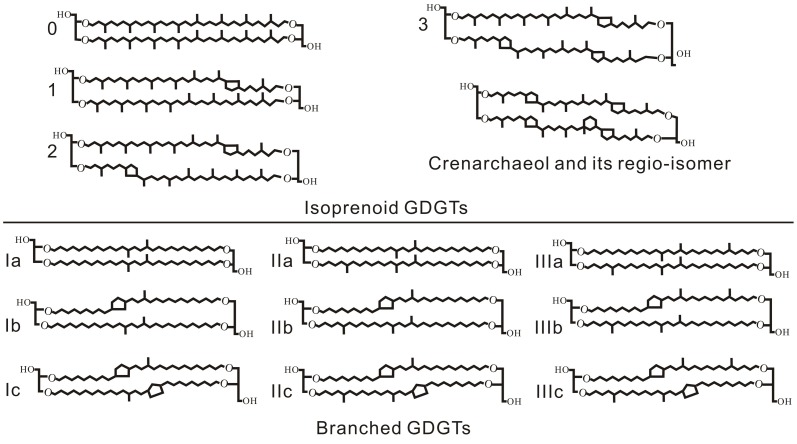
**Molecular structures of isoprenoid GDGTs and branched GDGTs**.

The microbial membrane lipids glycerol dialkyl glycerol tetraethers (GDGTs) are widespread, but different in structure, in archaea and bacteria (Figure 1). Archaea have isoprenoidal hydrocarbon chains, containing 0–3 cyclopentyl moieties or a cyclohexyl moiety in addition to four cyclopentyl moieties (i.e., crenarchaeol), at the *sn* 2,3 positions between the glycerol backbones (Schouten et al., 2000; Sinninghe Damsté et al., 2002). Whereas some specific bacteria have branched alkyl chains with 4–6 methyl groups, containing up to two cyclopentyl moieties, at *sn* 1,2 positions between the glycerol backbones (Weijers et al., 2006a).

Both the archaea and bacteria produced GDGTs have been used to develop temperature proxies that are promising and valuable in paleoclimatic studies. For example, sea surface temperatures were found being recorded by the distribution of isoprenoidal GDGTs (i-GDGTs) of marine Thaumarchaeota with a varying number of clyclopentane moieties, and thus a proxy called TEX_86_ (TetraEther Index of tetraethers having 86 carbons) has been developed (Schouten et al., 2002). TEX_86_ has also been used to estimate lake water temperatures in large lakes that receive minimal soil runoff (Blaga et al., 2009; Powers et al., 2010). Soon after, a proxy for continental air temperatures and soil pH was also proposed based on the distribution of soil bacterial branched GDGTs (br-GDGTs) by the definition of two indices, the methylation of br-GDGTs (MBT) and the cyclisation of br-GDGTs (CBT), as follows (Weijers et al., 2007):

(1) MBT=[Ia+Ib+Ic]/[Ia+Ib+Ic+IIa+IIb+IIc+IIIa+ IIIb+IIIc]

(2) CBT=−LOG([Ib+IIb]/[Ia+IIa])

The numbers in the equations refer to the abundance of the molecules in Figure 1. The soil pH and mean annual air temperature (MAT) can be then estimated by the following relationship:

(3) $$\text{CBT}=3.33-0.38*\text{pH}\left(n=114;r^{2}=0.70\right)$$

(4) $$\text{MBT}=0.122+0.187\;\times \;CBT+0.020\times \text{ MAT}\left(n=114;\;r^{2}=0.77\right)$$

Recently, the MBT index was adjusted according to a larger soil dataset (*n* = 278) as follows (Peterse et al., 2012):

(5) MBT′=[Ia+Ib+Ic]/[Ia+Ib+Ic+IIa+IIb+IIc+IIIa]

in which compounds IIIb and IIIc are not present, as in Equation (1), due to their undetectable amounts in most soil samples. As a result, a new calibration, generally in better agreement with independent proxy data, was proposed (Peterse et al., 2012):

(6) $$\text{MAT}\prime =0.81-5.67\;\times \text{ CBT}+31.0\;\times \text{ MBT}\prime \left(n=176;\;r^{2}=0.59\right)$$

The relative concentration of br-GDGTs and i-GDGTs in sediments and soils, potentially reflecting the relative br-GDGT producing bacteria and archaea input, is also a promising environmental indicator. Hopmans et al. (2004) introduced the Branched and Isoprenoid Tetraether (BIT) index for tracing soil organic matter (OM) input into a marine system as follows:

(7) BIT=[I+II+III]/[I+II+III+Cren]

where I, II, and III are the abundances of br-GDGTs without cyclopentane moieties and Cren is the abundance of crenarchaeol in a given sample (structures in Figure 1). The BIT index therefore ranges from 0 to 1. A value of 0 indicates the presence of only crenarchaeol with no br-GDGT inputs, and a value of 1 indicates the presence of only br-GDGTs, which was thought initially to represent marine OM and soil OM, respectively (Hopmans et al., 2004). However, subsequent works showed that crenarchaeol can be produced in small to moderate amounts in soils (Leininger et al., 2006; Weijers et al., 2006a,b; Walsh et al., 2008) and that br-GDGTs may also be produced *in situ* in aquatic environments (Peterse et al., 2009; Sinninghe Damsté et al., 2009; Tierney and Russell, 2009; Tierney et al., 2011; Zhu et al., 2011; Wang et al., 2012; Zhang et al., 2012). As an extreme example, in highly alkaline soils (pH > 7.5) under severe drought conditions, archaeal i-GDGTs with crenarchaeol as the main component were found predominant over bacterial br-GDGTs, as indicated by the increase in the abundance ratio of i-GDGTs to br-GDGTs, i.e., R_i/b_, to values as high as 6 at higher pH values (Xie et al., 2012). So the BIT Index might be difficult to accurately trace soil OM in aquatic environments under drought conditions. Nevertheless, the BIT Index, as well as R_i/b_ value, may potentially be used to trace variations in microbial structure in terms of archaea versus br-GDGTs producing bacteria, as results of soil environmental changes.

**Figure 2:**
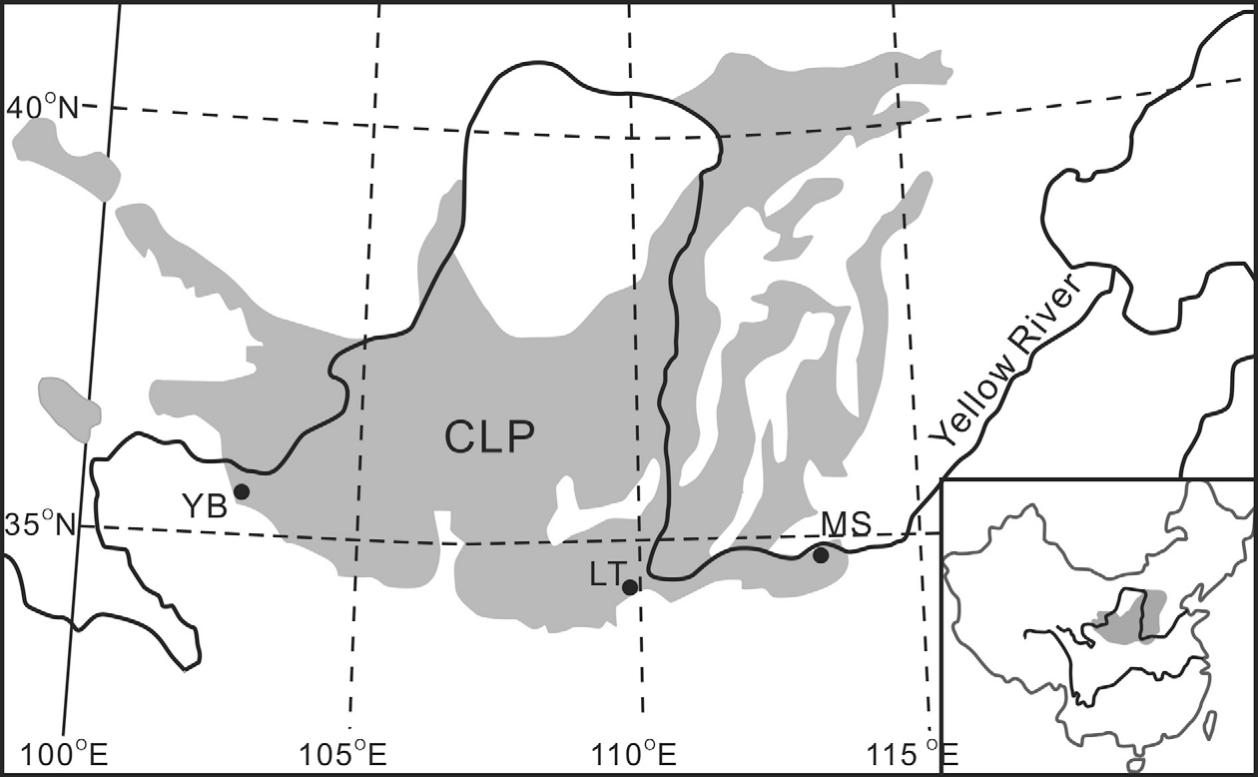
**Chinese Loess Plateau (CLP) and the site of Yuanbao (YB) in this study**. Sites LT (Lantian) and MS (Mangshan) have been studied for MBT-CBT temperature records by others and are mentioned in this paper.

The ubiquitous occurrence of GDGTs in soils offers the prospect of independently reconstructing continental paleotemperature from terrestrial deposits, e.g., the loess-paleosol sequences (LPS) from the Chinese Loess Plateau (CLP) (Figure 2). The LPS resulted from changes in monsoon intensity; loess is mainly deposited during cool and dry periods when East Asian winter monsoon (EAWM) intensifies, whereas soil development takes place during the warmer and wetter periods when East Asian summer monsoon (EASM) builds up (An, 2000). Although various climate proxies, e.g., pedogenic magnetic susceptibility (MS), phytoliths, grain size distributions and δ^18^O of rhizoconcretions and land snail shells, have been applied to unravel the history of the East Asian monsoon for the past several million years (e.g., Maher et al., 1994; Xiao et al., 1995; Li et al., 2007; Lu et al., 2007), little is known about the temperature history in the CLP region until the successful application of br-GDGTs for temperature reconstruction on the southeast CLP recently (Peterse et al., 2011; Gao et al., 2012). However, these works are clearly insufficient to reveal the whole picture of the temporal and spatial paleotemperature distributions with regard to the vast area of the CLP. In addition, failure examples of applying MBT-CBT proxy to three well-studied LPS in Serbia, northeast Siberia, and on Mt. Kilimanjaro prompt us to be cautious when interpreting these indices in LPS studies (Zech et al., 2012).

In this study, we present GDGTs results for the past 70 kyr from the Yuanbao LPS, western CLP. In addition to MBT-CBT temperature reconstruction and its comparison to previous results, records of BIT and R_i/b_ indices are also reported and discussed, which were not included in previous works.

## Materials and methods

### Site and sampling

**Figure 3:**
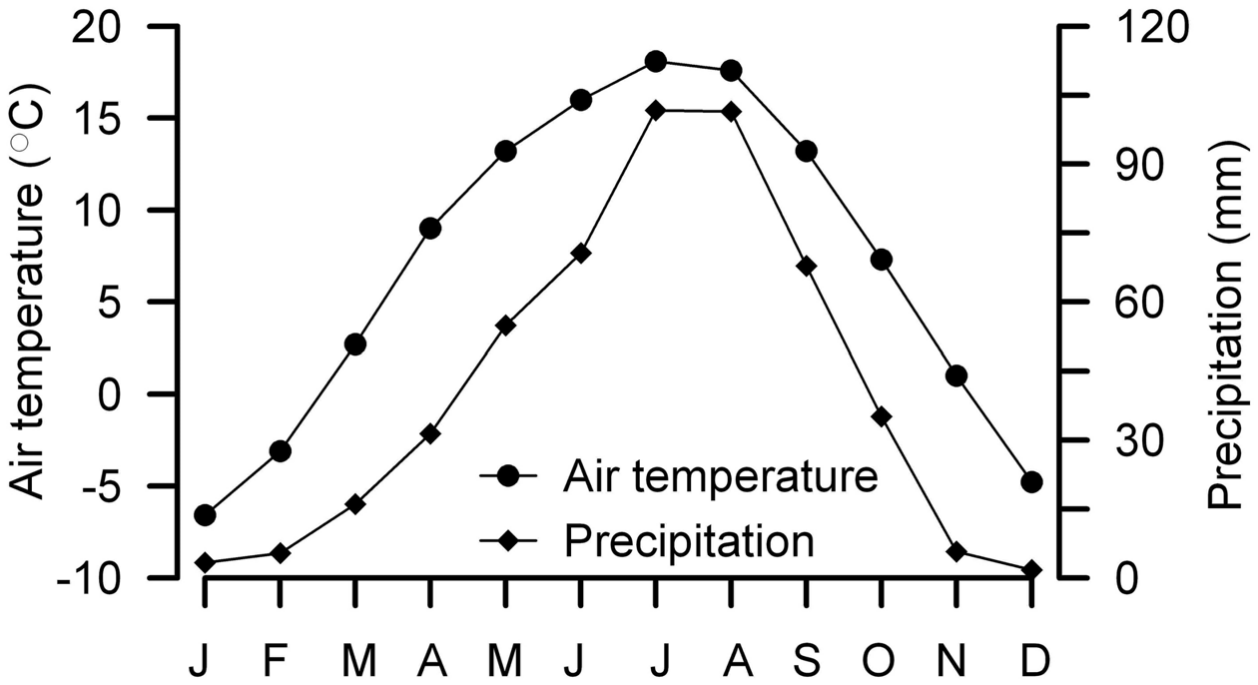
**Monthly distributions of temperature and precipitation between 1971 and 2000 at the Linxia city**. Data are from Public Meteorological Service Center, China Meteorological Administration. Note that the Yuanbao section is about 500 m above the Linxia city.

The site of Yuanbao sequence (103°09′E, 35°38′N) is located near the Linxia city in the western edge of the CLP prevailing with a typical temperate semi-arid climate (Figure 2) (Chen et al., 1997). The MAT at the Linxia city is ~7.0°C, with winter temperature below freezing (DJF) (Figure 3). The precipitation is concentrated from late spring to early autumn (May to September), accounting for 80% of the annual mean of 500 mm (Figure 3); whereas the annual evaporation is 1300 mm. The study site lies on the fourth terrace of the Xiahe River with an elevation of 2177 m, which is about 500 m above the Linxia city. According to the lapse rate of 0.6°C/100 m, the MAT at the site is estimated to be ~4°C. The loess on the river terrace is up to 150 m thick.

In the present study, we investigated the upper 27 m of the Yuanbao sequence from the bottom of loess L_1_ to the paleosol S_0_ with the top sample 0.5 m below the surface. The sequence was spliced by two exposed sections, CH02/02 and CH02/03, with 2–3 km apart (Chen et al., 1997, 1999; Lai and Wintle, 2006; Lai et al., 2007). The two sections have been dated by using high resolution optically stimulated luminescence (OSL), showing that the 4.0-m long section CH02/02 have been deposited since ~17.3 kyr before present (BP) and the interval from 17 m up to 0.5 m in the section CH02/03 was deposited from 51.2 to 17.5 kyr BP (Lai and Wintle, 2006; Lai et al., 2007). The exposed outer layers of the sections were removed during the course of sampling to ensure fresh samples for this study. A total of 40 samples, 15 in the section CH02/02 and 25 in the section CH02/03 were collected. The uppermost 0.5 m of in the CH02/02 and 1.1 m of the CH02/03 section are disturbed by ploughing, and thus were not sampled.

### Magnetic susceptibility, bulk organic carbon and soil pH analysis

Samples were oven dried at 50°C, lightly ground and aliquots of ~10 g were analyzed using a Bartington MS2 for MS. Total organic carbon (C_org_) was analyzed in duplicate using an elemental analyzer (Vario Pyro Cube) after removal of carbonate with diluted HCl. The average standard deviations of C_org_ is ±0.2%. The pH of the paleosol and loess samples was measured in a 1:2.5 soil:water (w/v) mixture using a digital pH meter.

### GDGT analysis

All air-dried samples, with each ca. 50 g in weight, were homogenized with a mortar and pestle, and ultrasonically extracted (9 ×) with MeOH (3 ×), dichloromethane (DCM)/MeOH (1:1, v/v; 2 ×) and DCM (2 ×) and all extracts were combined after centrifugation. Known amounts of an internal C_46_ GDGT standard were added according to Huguet et al. (2006). The total extracts were dried by way of rotary evaporation under vacuum and then were purified and separated into an apolar fraction and a polar fraction over an activated silica gel column by elution with *n*-hexane and DCM/MeOH (1:1, v/v), respectively, the GDGTs being in the polar fraction. The solvent was removed under N_2_ and the residue dissolved via sonication (5 min) in hexane/propanol (99:1, v/v) and filtered through a 0.45 μm, 4 mm diameter PTFE filter.

Analysis of GDGTs was performed using an Agilent 1200 HPLC/6410 TripleQuad MS instrument equipped with an auto-injector and Chemstation chromatography manager software. Procedures described by Schouten et al. (2007) were applied. Separation was achieved with a Prevail Cyano column (2.1 × 150 mm, 3 μm diameter; Grace, USA), maintained at 30°C. Injection volume varied from 10 to 20 μ l. GDGTs were eluted isocratically with 99% hexane and 1% propanol for 5 min, followed by a linear gradient to 1.8% propanol in 45 min. Flow rate was 0.2 ml/min. Detection was achieved using atmospheric pressure chemical ionization mass spectrometry (APCI-MS) via selected ion monotoring (SIM) of [M + H]^+^ ions (in MS1) and GDGTs were quantified by integration of the peak areas. Absolute quantification was achieved by calculating the area of the corresponding peaks in the chromatograms, comparing them with the peak area of the internal standard.

## Results and discussion

### Age model, magnetic susceptibility and C_org_ content

Age determination was according to the OSL dating results on the sections CH02/02 and CH02/03 (Lai and Wintle, 2006; Lai et al., 2007). For the section CH02/02, our MS curve was correlated with that from Lai and Wintle (2006) before age determination instead of applying the given depth-age relationship, which may reduce the errors from depth measurement difference between the two studies. For the section CH02/03, because no MS data were presented along with age determination in Lai et al. (2007), the depth-age relationship (Lai et al., 2007) was applied to our samples.

**Figure 4:**
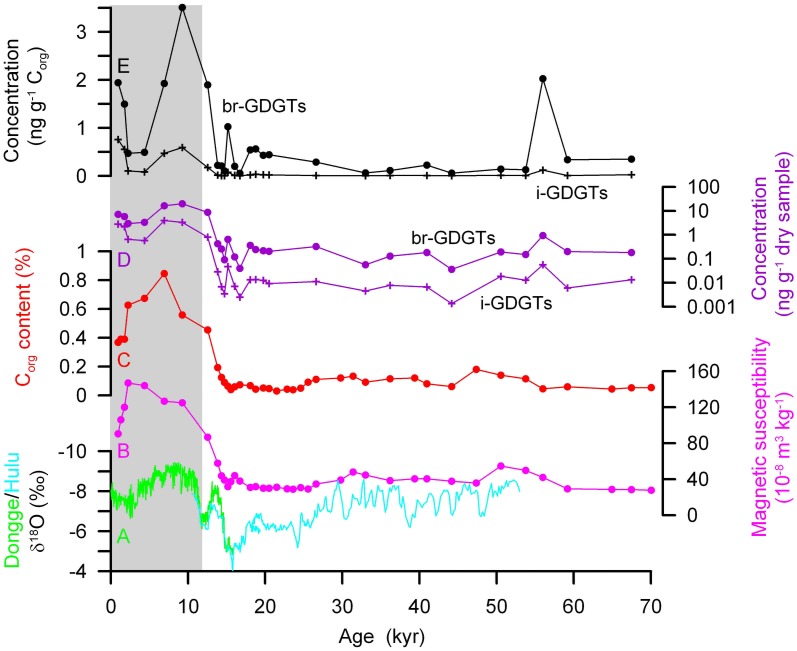
**Variations down the loess-paleosol sequence at Yuanbao section of (B) magnetic susceptibility, (C) C_org_ content, (D) concentrations of i-GDGTs and br-GDGTs in dry samples, and (E) C_org_-content normalized concentrations of i-GDGTs and br-GDGTs**. Speleothem δ^18^O **(A)** indicating East Asian summer monsoon (Wang et al., 2001; Dykoski et al., 2005) is applied for comparison. The gray area denotes the Holocene period since ~12 kyr BP, and the area before that denotes the last glacial time.

Based on the age model, MS and C_org_ (wt%) content in the Yuanbao sequence changed in parallel, with lower values during the glacial time, the deglacial onset of increases at ~15 kyr BP, and higher values during the Holocene (Figures 4B,C). The two records are similar to the changes of speleothem δ^18^O (Figure 4A), as is expected because all of these proxies are associated with the development of the EASM precipitation (Zhou et al., 1990; Wang et al., 2001; Dykoski et al., 2005; Peterse et al., 2011). However, there is an obvious asynchrony between the peaks of MS (at 2.2 kyr BP) and the C_org_ content (at 6.9 kyr BP). This mismatch in time might be due to that MS is a complex climate proxy and its value is not always consistent with the soil development, which has been pointed out by Guo et al. (1998). The low sampling resolution might be also responsible for the mismatch. But overall, records of MS and C_org_ suggest that EASM was strongest during the mid Holocene, consistent with numerous results on the regional paleoclimate [reviewed by (Zhao et al., 2009)]. A fine comparison of the timing of EASM development with other records, e.g. the Mangshan record, is difficult at present, because of the low sampling resolution in this study and the different dating methods for age models. However, the onset of EASM enhancement seems synchronous across CLP, as both MS and C_org_ began to rise at ~15 kyr BP in both Yuanbao and Mangshan sequences.

### GDGTs concentration and distribution

A remarkable feature of GDGT concentration profiles in the Yuanbao LPS is the large differences in their concentration and distribution between the last glacial loess and the Holocence paleosol (Supplementary data sheet).

Br-GDGTs are present in all paleosols, but they were detected in only 19 out of 31 loess samples. Their total concentration in the paleosols varies between 2.9 and 19.6 ng g^−1^, whereas it is much lower in loess samples with values between 0.04 and 0.9 ng g^−1^ (Figure 4D). However, if normalized to TOC content, the difference between loess and paleosol becomes reduced, i.e., 0.5–3.5 μg g^−1^ TOC in paleosols and 0.05–2.0 μ g g^−1^ TOC in loess (Figure 4E). In addition to the concentration difference, loess samples do not contain detectable amounts of all nine br-GDGTs that are used for the CBT and MBT calculations, as paleosols do. In particular, br-GDGTs Ic, IIc, and IIIc in loess samples are either completely undetectable (i.e. br-GDGT IIIc) or frequently below detection limit and, if present, less than 1% of total br-GDGTs on average (i.e., br-GDGTs IC and IIC). The most dominant br-GDGTs in the LPS is IIa, accounting for 33.2% (23.4–45.2% range) in total br-GDGTs. Br-GDGT Ia, IIIa, Ib and IIb are present in all samples, but with smaller percentages (3.6–25.1% range). Br-GDGT IIIb is less than 8.4% and in several loess samples it is below detection limit.

**Figure 5:**
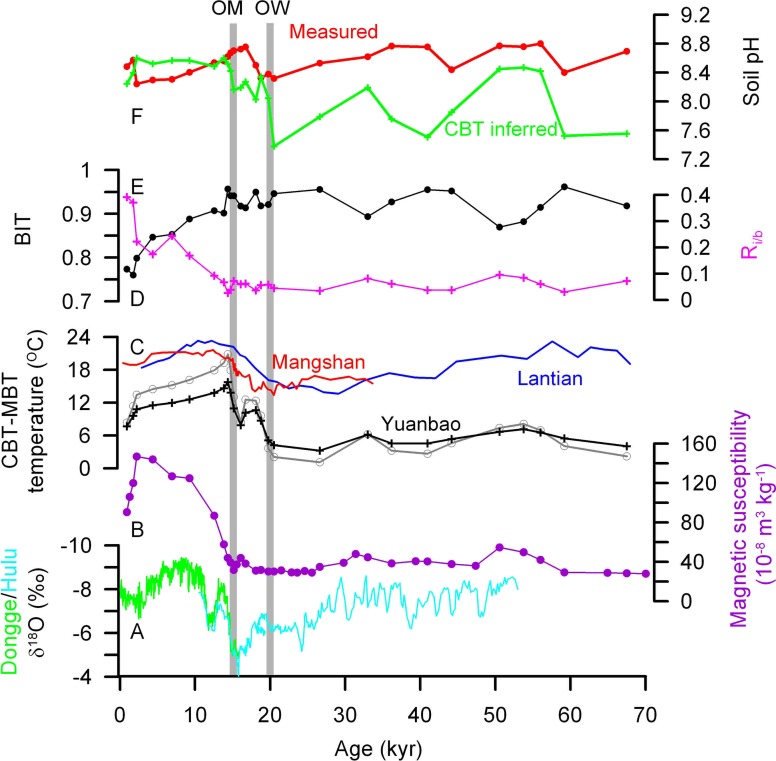
**GDGT derived proxy records down the sequence at Yuanbao section and their comparison to speleothem δ^18^O**. **(A)** Speleothem δ^18^O from Dongge and Hulu cave (Wang et al., 2001; Dykoski et al., 2005); **(B)** magnetic susceptibility; **(C)** MBT-CBT derived temperature. Black and gray curves are from Yuanbao sequence using Equations (6) and (4), respectively; red and blue curves are from Manshan (Peterse et al., 2012) and Lantian sequences (Gao et al., 2012), respectively; **(D)** BIT; **(E)** R_i/b_; **(F)** Soil pH. OM: onset of East Asian summer monsoom; OW, onset of warming.

I-GDGTs also occur, although in less amounts, in the sequence. Their total concentrations are higher in paleosols (0.6–4.0 ng g^−1^ dry bulk sample, or 0.09–0.76 μg g^−1^ TOC) than in loess (0.001–0.06 ng g^−1^ dry bulk sample, or 0.002–0.12 μg g^−1^ TOC) (Figures 4D,E). All i-GDGTs in Figure 1 are present in all the paleosols with crenarchaeol the dominant component (40.5–65.7% range); however, crenarchaeol becomes the only i-GDGT compound in the loess except two samples. This suggests that archaeal community structure in the glacial loess might be quite different from that in the Holocene paleosols, with the crenarchaeol-producing Thaumarchaeota predominant during the last glaciation. The cause for the Thaumarchaeota predominance in archaeal community might be related to the slightly more alkaline conditions during the glacial time, as indicated by the higher pH values measured in loess samples (Figure 5F), under which elevated ammonia oxidation rate may promote the growth rate of soil ammonia-oxidizing Thaumarchaeota (Xie et al., 2012).

The occurrence of higher br-GDGT concentration in paleosol than in loess has been also observed in the Mangshan sequence (Peterse et al., 2011), although not observed in the Lantian sequence (Gao et al., 2012). Higher concentrations of GDGTs, including both i-GDGTs and br-GDGTs, in paleosol suggest that microbial activity is an important agent in the pedogenic processes. Likewise, the higher TOC normalized concentrations of GDGTs in paleosols could indicate greater contributions of microbial OM to the total OM. However, although GDGTs were not detected in some loess samples in this study, they were present in the loess layers at the Mangshan and Lantian sequences (Peterse et al., 2011; Gao et al., 2012). Two factors during the last glacial time might be responsible for the discrepancy: (1) Temperature was lower, and perhaps aridity was higher, at Yuanbao than at Mangshan and Lantian, which was more unfavorable for microbial and biomass growth. (2) Dust accumulation rate was several time higher in the NW CLP than in the SE CLP (Lai et al., 2007). So GDGTs concentrations could have been more diluted in the Yuanbao loess layers, and thus are hard to be detected unless loess sample amounts are added.

### Temperature record

Although atmospheric dust from the loess plateau has not been analyzed, samples in loess source regions and a dust sample from near the west coast of Central Africa have been found containing negligible amounts of GDGTs (Hopmans et al., 2004; Gao et al., 2012). Thus, GDGTs in the LPS are likely locally produced, and the MBT-CBT record reflects local conditions (Peterse et al., 2011; Gao et al., 2012).

Due to lack of surface soil sample, temperature estimate of ~8.3°C from Equation (4) or 7.6°C from Equation (6) for the youngest sample (i.e., ~0.9 kyr BP) is compared to present. It is higher than present MAT of ~4°C, but lower than summer (June-August) mean of ~14°C at the Yuanbao site. In the Lantian sequence, temperature estimate of ~18.4°C from Equation (4) for the youngest sample at 2.9 kyr BP also fell in between the local MAT of 13.1°C and summer mean of 26°C (Gao et al., 2012). In the Mangshan sequence, estimate of ~23°C from Equation (4) for samples for the last 2 kyr was in agreement with local summer temperature (Peterse et al., 2011). Reconstructed MATs bias to local warm season temperatures have been also observed in some investigations on sediments from lakes, coastal area and peat bog (Rueda et al., 2009; Huguet et al., 2010; Sun et al., 2011). Thus it is likely that the GDGT-producing bacteria might thrive in the warm season of a year. However, in a 1-year investigation on soils sampled from eight different plots in the mid-latitude USA, The Netherlands and the UK, the MBT-CBT temperature proxy did not show any seasonal patterns due to the fact that branched GDGT core lipids represent a standing stock that has accumulated over the course of years (ca. 20 years, Weijers et al., 2011). In that investigation, the authors also found that the MBT-CBT temperature, though calibrated with annual MAT, did not always exactly reflect the MAT, which resulted from the offset between soil and air temperature (Weijers et al., 2011). Therefore, the higher than global mean offset between soil and air temperature in the CLP region might be the cause of higher MBT-CBT temperature than the local MAT observed here and in previous works (Peterse et al., 2011, 2012; Gao et al., 2012). Nevertheless, the MBT-CBT proxy is still thought to be able to provide reasonable estimates of past changes of MAT, although the reconstructed absolute temperature is associated with a slightly larger error (ca. 5°C) (Weijers et al., 2011).

The MBT-CBT derived temperature calculated by application of Equation (4) for the Yuanbao sequence varies between 1.1°C and 20.8°C. Temperature was <8.1°C in the last glacial time between 70 and 20 kyr BP, and then a deglacial warming occurred from 3.7°C at 19.7 kyr BP to 20.8°C at 14.3 kyr BP, the highest value in the time frame (Figure 5C). The Holocene is featured by a continuous cooling trend towards a temperature value of 8.3°C for the top sample. Since br-GDGT IIIc and IIIB are either undetectable or a minor component in our samples, Equation (5) was also used to recalculate the MBT-CBT derived temperature. The air temperatures that are derived with the new calibration are lower than the original record for most samples, but higher for several loess samples in the last glacial time (Figure 5C). Thus, the new record suggests a smaller increase in air temperature over the deglaciation with temperature estimates increasing from 5.1°C to a maximum of 15.8°C.

The MBT-CBT derived temperature records from other LPSs also suggested a large increase in air temperature over the deglaciation, e.g., from 13.5°C at 20.5 kyr BP to a maximum of 27.2°C at 12.4 kyr BP in the Mangshan sequence and from 14.6°C at 22.5 kyr BP to a maximum of 23.3°C at 12.2 kyr BP in the Lantian sequence (Peterse et al., 2011; Gao et al., 2012). By using the new calibration (6), Peterse et al. (2012) gave a smaller deglacial warming of 6 ~ 7°C for the Mangshan sequence (Red curve in Figure 5C). Our temperature record shows a deglacial increase of ~18°C or ~10°C by application of Equation (4) or (6), respectively. The deglacial increase of ~10°C from Equation (6) seems more reasonable, as 4.5–9°C cooler air temperatures during the LGM and 1–2°C warmer during the Holocene climatic optimum than at present in central Asia have been estimated based on pollen and phytolith studies (Sun et al., 1997; Lu et al., 2007). The lower absolute temperature estimates and larger deglacial warming at Yuanbao than those at Mangshan and Lantian may be due to the higher latitude and farther inland of the Yuanbao site.

Similar to the findings of early warming on the SE CLP relative to EASM precipitation increase by Peterse et al. (2011) and Gao et al. (2012), our record also suggests early warming in the western CLP, as indicated by the lead of onset of warming at ~20 kyr BP to the onset of MS and C_org_ increase at ~15 ky BP from the same sequence (Figures 5A–C). The high temperature and still low EASM precipitation during the early Holocene is in agreement with the finding that wind-blown sands were mobile at many sites along the desert margin in northern China due to hot and dry conditions (Mason et al., 2009). Therefore, although the EASM precipitation history on its temporal and spatial distributions are still in debate (An et al., 2000; Zhao et al., 2009), the early deglacial warming was likely regionally synchronous across the CLP.

### Br-GDGTS vs. i-GDGTs

The BIT index exhibits higher values (0.87–0.96 range, 0.93 average) in the glacial loess and lower values (0.76–0.91 range, 0.83 average) in the Holocene paleosols (Figure 5D). Similar occurrence, but in opposite direction, is also shown in the record of R_i/b_ that indicates the relative abundance of total i-GDGTs to total br-GDGTs (Figure 5E). As the production of both archaea and br-GDGT producing bacteria could have increased in the pedogenic process during the Holocene, the records of BIT and R_i/b_ could suggest that the increase of archaea might have exceeded that of the br-GDGT producing bacteria. The increase of R_i/b_ has been proposed to indicate alkaline soils induced by enhanced aridity (Xie et al., 2012), perhaps due to that soil Thaumarchaeota may produce higher amounts of crenarchaeol under alkaline conditions (Weijers et al., 2006b; Pearson et al., 2008). Indeed, CBT-derived pH record shows averagely higher values (~8.5 ± 0.1) during the Holocene than during the last glciation (~8.1 ± 0.4) (Figure 5F). However, the pH record is not consistent with what was obtained from direct pH measurement on samples, showing slightly higher pH during the last glaciation. This disagreement is not clear at present, but might reflect the difficulty of the CBT index to precisely estimate alkaline soil pH (Xie et al., 2012). In addition, the increasing trend in R_i/b_ record since the early Holocene is not in accordance with the unchanged CBT-derived pH record, or even in opposite direction to the directly measured pH record. Therefore, the R_i/b_ record in this study seems unlikely to be caused by soil pH changes.

We notice that the steadily increasing (or increasing) trend of R_i/b_ (or BIT) since the early Holocene matches well with the MBT-CBT temperature record (Figure 5C). However, it seems unlikely to establish a causal relationship between them, because other soil physicochemical conditions in addition to temperature, such as moisture, pH, redox conditions and nutrient levels, might be also important to influence the microbial community. Soil physicochemical conditions should have been associated more closely with precipitation and OM accumulation, which can be traced by MS and TOC records in this study. However, the BIT and R_i/b_ records for the Holocene do not match well with those of MS and C_org_.

Alternatively, the decreasing (or increasing) trend of BIT (or R_i/b_) since the early Holocene may be caused by the differential degradation of i-GDGTs vs. br-GDGTs. If so, the trend would suggest that i-GDGTs were preferentially degraded and/or br-GDGTs were selectively preserved in the sediment sequence. This inference is in agreement with observations from marine sediment analysis (Huguet et al., 2008, 2009) and modeling experiments (Ding et al., 2013). For example, Huguet et al. (2008) indicated that degradation rates of crenarchaeol are 2-fold higher than those of soil-derived br-GDGTs. Besides, there are also other potential unrealized occurrences associated with the secular BIT and R_i/b_ changes. For example, we do not know the depth distribution of living archaea and GDGT-producing bacteria in soils, i.e., the growth depth effect as proposed by Zech et al. (2012). Therefore, upward increase in living archaea relative to bacteria in the paleosol profile cannot be fully excluded. This issue may be resolved by analyzing intact polar GDGTs, markers for living cells, in the profile in future. In any case, it reminds us to be cautious with BIT and R_i/b_ proxies in paleo-environmental reconstructions.

## Conclusions

The Yuanbao loess-paleosol sequence from the west CLP shows clear biogeochemical and climate contrasts between the last glaciation and the Holocene. Higher C_org_ content and GDGT concentrations in the Holocene paleosols consistently indicate an intensified pedogenic process as results of strengthened warm and wet EASM. MBT-CBT derived temperature record displays an early deglacial warming relative to the onset of EASM, which is in agreement with previous findings from the southeastern CLP and suggests that the early deglacial warming was likely regionally synchronous across the CLP. In the BIT (or R_i/b_) variations, a steady decrease (or increase) since the early Holocene is prominent. Changes in microbial community, i.e., archaea production exceeding that of the br-GDGT producing bacteria, could be responsible for this. However, other possibilities, such as preferential degradation of isoprenoid GDGTs or upward increase in living archaea relative to bacteria in the paleosol profile, cannot be fully excluded. Thus, future studies, with foci on microbial community structure in soil column and differential degradation of GDGT molecules, are needed for accurate paleoclimate and paleo-biogeochemical reconstructions from the CLP.

### Conflict of interest statement

The authors declare that the research was conducted in the absence of any commercial or financial relationships that could be construed as a potential conflict of interest.
